# In vitro anti-adenoviral activities of ethanol extract, fractions, and main phenolic compounds of pomegranate (*Punica granatum* L.) peel

**DOI:** 10.1177/2040206620916571

**Published:** 2020-04-19

**Authors:** Ali Karimi, Mohammad-Taghi Moradi, Mohammad Rabiei, Somayeh Alidadi

**Affiliations:** 1 Cellular and Molecular Research Center, Basic Health Sciences Institute, Shahrekord University of Medical Sciences, Shahrekord, Iran; 2 Medical Plants Research Center, Basic Health Sciences Institute, Shahrekord University of Medical Sciences, Shahrekord, Iran; 3 Department of Pathobiology, Infectious Disease and Public Health, School of Animal and Veterinary Sciences, University of Adelaide, Adelaide, Australia; 4 Clinical Biochemistry Research Center, Basic Health Sciences Institute, Shahrekord University of Medical Science, Shahrekord, Iran

**Keywords:** Antiviral, Punica granatum L., crude extract, compounds, fractions, adenovirus

## Abstract

**Background:**

Adenovirus causes a number of diseases in human, and can cause serious infection in severely immunosuppressed individuals. Despite the seriousness of adenovirus infection, there is no definitely approved anti-adenoviral therapy. Many studies have shown that compounds derived from medicinal plants have antiviral activity. Therefore, this study evaluated in vitro anti-adenoviral activity of ethanol extract, fractions, and main phenolic compounds of pomegranate peel.

**Methods:**

The ethanol extract of pomegranate peel was prepared with maceration method and fractionated by consecutive liquid/liquid partition. The cytotoxic and anti-adenovirus activities of the extract, fractions, and main phenolic compounds (ellagic acid, punicalagin and gallic acid) were evaluated on Hep-2 cell line using MTT assay. Inhibitory effect on adsorption and post-adsorption phases of the virus replication cycle was also evaluated.

**Results:**

Pomegranate peel extract had a desirable effect against adenovirus with IC_50_ of 5.77 µg/mL and selectivity index of 49.9. Among the fractions and compounds, the *n*-butanol fraction and gallic acid had the highest anti-adenoviral activity with IC_50_ of 2.16 µg/mL and 4.67 µM and selectivity indices of 122.5 and 10.5, respectively. The crude extract, *n*-butanol fraction and gallic acid inhibited the virus replication in post-adsorption phase (*p *<* *0.01).

**Conclusion:**

Pomegranate peel extract, especially its *n*-butanol fraction, could serve as a new promising anti-adenovirus agent due to high inhibitory effect against adenovirus replication. The effect of the *n*-butanol fraction may be related to the synergistic effect or other compounds of this fraction. Further understanding of the bioassay guided isolation of natural compounds of this fraction seems essential.

## Introduction

Adenoviruses (ADVs) are non-enveloped double-stranded DNA viruses that are of over 70 types (genotypes). Human ADVs are classified into seven species (A–G). Generally, human ADVs are not highly pathogenic, and are mostly associated with self-contained respiratory infections, hemorrhagic cystitis, and gastroenteritis, particularly in infants and young children.^1^ In addition, ocular human ADV infections are among the leading causes of viral conjunctivitis. In immunocompromised patients, such as solid-organ transplant or hematopoietic stem cell transplant recipients, human ADV infections leaf to high morbidity and mortality.^2^ In fact, human ADV is reportedly the most increased virus in transplant recipients, especially in pediatric units.^3^

No effective therapy or definitely approved drug has yet been developed for ADV associated diseases. Cidofovir and ribavirin are occasionally used in clinics. Although no controlled clinical trial has yet been conducted on the two drugs, they appear to exhibit activity against ADV.^4^ Two problems with cidofovir are severe central nervous system side effects and retinal toxicity.^4,5^ Because of the lack of effective anti-adenoviral drugs, it is necessary to develop alternative anti-adenoviral treatments.

Attempts are being made to develop various novel agents that may be effective against viruses, specifically human ADV. Naturally occurring anti-viral nutrients may be of special importance because they are widely available and may be used as a constituent of the diet to fight diseases, including ADV infection.

The pomegranate, botanically called *Punica granatum*, belongs to the Punicaceae family. In traditional medicine, pomegranate fruit is used to relieve hepatic failure, dry coughing, facial swelling, skin itching, and jaundice. Pomegranate peel is also useful for the treatment of sore throats, gastrointestinal worms, and diarrhea.^6^ Metabolites in different parts of the fruit and peel of pomegranate contain a variety of sugars, organic acid, alkaloids, polyphenols, tannins, flavonoids, anthocyanins, fatty acids, vitamins, etc.^6,7^ Pomegranate peel is a rich source of tannins and other phenolic compounds.^8^ Chidambara Murthy et al. have also reported that pomegranate peel contains ellagic acid, ellagitannins, and gallic acids.^9^

It has been observed that many plant polyphenols, such as ellagic acid, catechins, and chlorogenic, caffeic, and ferulic acids, act as antibacterial, antiviral, antioxidant, anti-inflammatory, and antineoplastic agents.^10–12^ In the present study, we prepared crude ethanol extract and four fractions of pomegranate peel, and then investigated the efficacy of these plant materials and three main phenolic compounds of pomegranate peel (ellagic acid, punicalagin and gallic acid) on human ADV.

## Materials and methods

### Chemicals and reagents

Ethyl alcohol, *n*-hexane, chloroform, ethyl acetate, *n*-butanol, and dimethyl sulfoxide (DMSO) were purchased from CARLO ERBA Reagents (France). Butylated hydroxytoluene, 2,2 diphenyl-1-picrylhydrazyl, gallic acid, ellagic acid, punicalagin, rutin, and 3-(4,5-dimethylthiazol-2ol) 2,5-diphenyl tetrazolium bromide (MTT) were purchased from Sigma–Aldrich (St. Louis, USA). Folin–Ciocalteu, aluminum chloride, potassium acetate, and sodium acetate were supplied from Merck Co. (Darmstadt, Germany). Dulbecco’s Modified Eagle’s Medium (DMEM), fetal bovine serum (FBS), penicillin-streptomycin, and amphotericin B were purchased from Gibco (USA).

### Plant collection, extraction, and fractionation

*P*. *granatum* of the *Malas* variant was obtained from Shahreza, Isfahan Province, Iran. The dried pomegranate peels were separately pulverized to obtain uniform powders. The peel powder (100 g) was dissolved in 80% ethyl alcohol (400 mL) and the resulting solution was stored at room temperature for 96 h. Then, the mixture was filtered and concentrated under nearly vacuum pressure at 40 °C in rotary evaporator. The extracts were stored in sterile bottles under refrigerated conditions until further use. The crude extract was dissolved in ethyl alcohol/H_2_O and fractionated by consecutive liquid/liquid partition with *n*-hexane and then with chloroform, ethyl acetate, and *n*-butanol with increasing polarity.^13^ The extract/fractions were suspended at 37 °C in 10% DMSO to yield a stock solution of 10 mg/mL. This solution was filtered (Millipore® 0.22 µm) and stored (at 4 °C) until used. A small percentage (up to 0.2%) of DMSO present in the wells has no effect on the results of the experiments.^13^

### Cell and virus

Hep-2 (human laryngeal epidermal carcinoma) cells were kindly provided by Pasteur Institute of Iran, Tehran, Iran. The cells were grown in DMEM supplemented with 10% of FBS, 100 µg/mL of streptomycin, 100 UI/mL of penicillin and 0.25 µg/mL amphotericin B at 37 °C with 5% CO_2_. The same medium containing 1.5% FBS was used for cytotoxicity and antiviral assays. ADV (type 5) was kindly provided by the Faculty of Health, Tehran University of Medical Sciences, Tehran, Iran. Virus stock was prepared by infection of 80% confluent monolayer Hep-2 cells in 75 cm^2^ culture flasks using DMEM with 1.5% FBS, at 37 °C in 5% CO_2_. Virus titer was determined by cytopathic effect (CPE) of ADV in Hep-2 cells and was expressed as the 50% tissue culture infective dose (TCID_50_) per mL.

### Cytotoxicity assay

For cytotoxicity assays, 50% cytotoxic concentrations (CC_50_) of the plant materials were determined using the MTT assay.^14^ Briefly, Hep-2 cells were seeded onto 96-well plates at a concentration of 8000 cells/well to a final volume of 100 µL per well. After incubation at 37 °C with 5% CO_2_ for 24 h, when the cell monolayer was 80% confluent, the cell culture medium of cells was aspirated and washed with phosphate-buffered saline (PBS). Cells were incubated with 200 µL/well of various concentrations of plant materials (in triplicate) and incubated at 37 °C with 5% CO_2_ for further four days. Cell viability was examined by ability of the cells to cleave the tetrazolium salt in the MTT assay using succinate dehydrogenase mitochondrial enzyme, which develops a formazan blue color product according to a procedure that has been described earlier.^14^ Briefly, the supernatants were removed from the wells and 50 µL of an MTT solution (1 mg/mL in PBS) was added to each well. The plates were incubated for 4 h at 37 °C, and 100 µL of DMSO was added to the wells to dissolve the MTT crystals. The plates were shaken on a shaker for 15 min and the absorbance was read by an enzyme-linked immunosorbent assay reader (STATA FAX 2100, USA) at 570 nm wavelength. Data were calculated by the following formula and expressed as the percentage of toxicity: toxicity (%) = (100 − (*A*_t_/*A*_s_) × 100)%, where *A*_t_ and *A*_s_ refer to the absorbance of the test substance and the solvent control, respectively.^13,14^ The CC_50_ was defined by nonlinear regression.

### Antiviral assay

Antiviral activity of the plant materials was evaluated by testing their inhibitory activity using the MTT assay according to a procedure described previously.^12^ Briefly, 100 µL (100 TCID_50_) of virus suspension was added to at least 80% of confluent HEp-2 cell monolayer in a 96-well plate and incubated at 37 °C for about 2 h to allow virus adsorption. Then, serial two-fold dilutions that had been prepared from non-toxic dose of the plant materials were added and the resulting solution was tested in triplicate. To prepare positive control, cells were infected with the same concentration of virus but without addition of plant materials. To prepare negative or cell control, only DMEM and 1.5% FBS were added to the cells. The plates were incubated at 37 °C in a humidified CO_2_ atmosphere for four days.

Cell viability was also determined using previously described MTT assay.^14^ Data were calculated and expressed as the percentage of inhibition using the following formula: antiviral activity (%) = (*A*_tv _− *A*_cv_)/(*A*_cd _− *A*_cv_) × 100%, where *A*_tv_, *A*_cv_, and *A*_cd_ represent the absorbance of the test compounds on virus-infected cells, the absorbance of the virus control and the absorbance of the cell control, respectively. The procedure was carried out in triplicate. The 50% inhibitory concentration (IC_50_) was determined from a curve relating inhibition to the concentration of the plant materials. The CC_50_/IC_50_ was calculated to determine selectivity index (SI), an index of antiviral activity.

### Mode of antiviral activity

**Figure 1.**
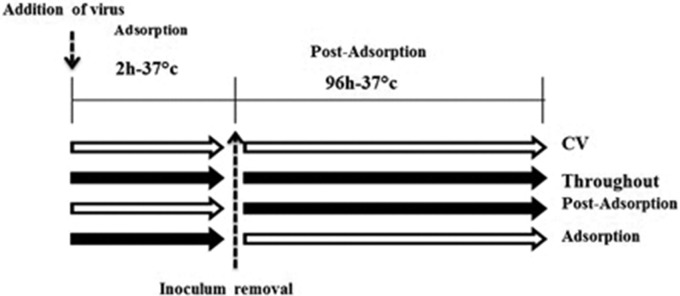
Scheme of addition of plant materials in the adsorption and post-adsorption phases of the virus. Open and black arrows indicate the absence and presence of plant materials, respectively.

To assess the mechanism of antiviral activity, the time-of-addition effect of the plant materials with better anti-adenoviral effects (crude extract, *n*-butanol fraction and gallic acid) was evaluated according to a previously described procedure with minor modifications.^15^ Infected cell cultures were treated with one time IC_90_ of the plant materials (crude extract 11.8 µg/mL, *n*-butanol fraction 4.1 µg/mL and gallic acid 12.4 µM) at three different times: (1) after adsorption and until the end of the experiment (post-adsorption); (2) during and after the adsorption (throughout); and (3) during the adsorption (adsorption; Figure 1). To conduct these experiments, 80% confluent cells were infected with 100 TCID_50_ (100 µL/well) of the virus in the presence or absence of plant materials and further incubated at 37 °C for 2 h so that only the adsorption step of the viral particles to the cells (adsorption) was performed. Then, the supernatant was replaced with the medium and 1.5% FBS with or without the plant materials, and then incubated for four days at 37 °C with 5% CO_2_. Cell viability and the percentage of virus inhibition were also evaluated compared with the control using the previously described MTT assay. The procedure was carried out in triplicate.

### Statistical analysis

All experiments were carried out in triplicate. The IC_50_ and CC_50_ were calculated using dose-response analyses and related models with probit procedure in the SPSS. Significance level (*p*) was considered <0.05.

## Results

### Cytotoxicity and antiviral assay

**Figure 2.**
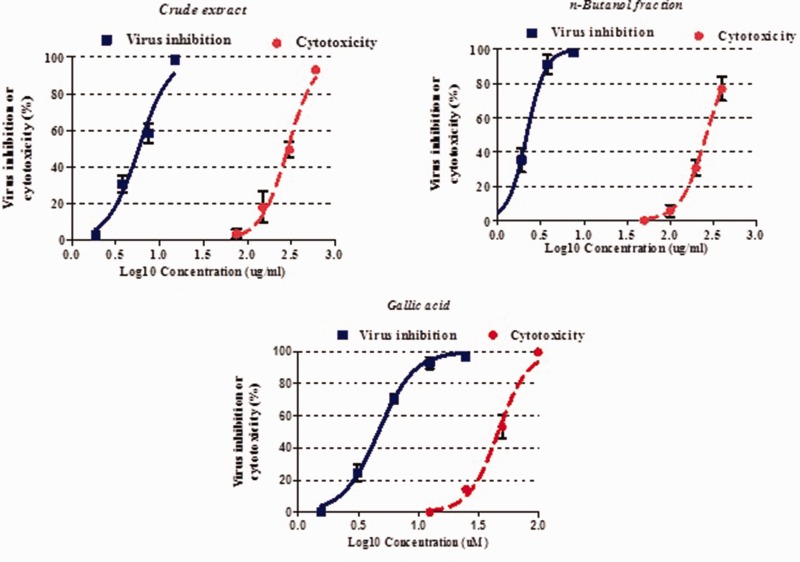
Cytotoxicity and anti-adenoviral activity of crude extract, *n*-butanol fraction, and gallic acid of pomegranate peel. Eighty percent confluent Hep-2 cells without virus infection or infected with virus were exposed to different concentrations of crude extract, *n*-butanol fraction, and gallic acid for 96 h. Cell viability was measured by the MTT test. Values are expressed as mean (±standard error of measurement) of three experiments.

Cytotoxic activities of plant extracts, fractions, and components were evaluated using Hep-2 cell line. The CC_50_ of pomegranate peel crude extract on Hep-2 cells was 288.2 µg/mL. The ethyl acetate fraction showed the highest cytotoxic activity with a CC_50_ of 32.4 µg/mL. Other fractions including *n*-butanol, chloroform, and *n*-hexane had a CC_50_ of 264.7, 151.4, and 125.9 µg/mL, respectively. Among the compounds, ellagic acid exhibited the highest cytotoxicity on the Hep-2 cells with a CC_50_ of 7.05 µm/mL (Table 1). The analysis demonstrated that the extract concentration was significantly associated with the cell death (*p *<* *0.05, Figure 2).

**Table 1.** Cell cytotoxicity, anti-adenoviral activity, and selectivity index of the crude extract, fractions, and main phenolic compounds of pomegranate peel.

Sample	CC_50_ (CI 95%)	IC_50_ (CI 95%)	SI
Crude extract (µg/mL)	288.2 (257.4–322.6)	5.77 (5.12–6.51)	49.95
*n*-Hexane fraction (µg/mL)	125.95 (90–164.5)	>125.95	<1
Chloroform fraction (µg/mL)	151.4 (128.5–178)	30.47 (3.27–58.1)	4.96
Ethyl acetate fraction (µg/mL)	32.4 (28.19–37.26)	5.41 (4.79–6.12)	5.98
n-Butanol fraction (µg/mL)	264.7 (234.9–298.4)	2.16 (1.94–2.41)	122.5
Gallic acid (µM)	49.34 (44.6–52.02)	4.67 (4.29–5.08)	10.5
Ellagic acid (µM)	7.048 (5.84–8.5)	>7.048	<1
Punicalagin (µM)	13.58 (1255–14.69)	4.48 (3.79–5.31)	3.03

CC_50_: 50% cytotoxicity concentration; IC_50_: 50% inhibitory concentration; CI 95%: confidence interval 95 ℅, SI: selectivity index.

The antiviral activity showed that pomegranate peel extract had a desirable effect against ADV with an IC_50_ of 5.77 (CI 95%: 5.12–6.51) µg/mL, and SI of 49.9. Out of the fractions and compounds, the *n*-butanol fraction and gallic acid had the highest anti-adenoviral activity with an IC_50_ of 2.16 (CI 95%: 1.94–2.41) µg/mL and 4.67 (CI 95%: 4.3–5.1) µM and a SI of 122.5 and 10.5, respectively (Table 1). Our results showed that the higher the extract concentration, the more pronounced the CPE inhibition (Figure 2, p < 0.05).

### Characterization of antiviral activity

**Figure 3.**
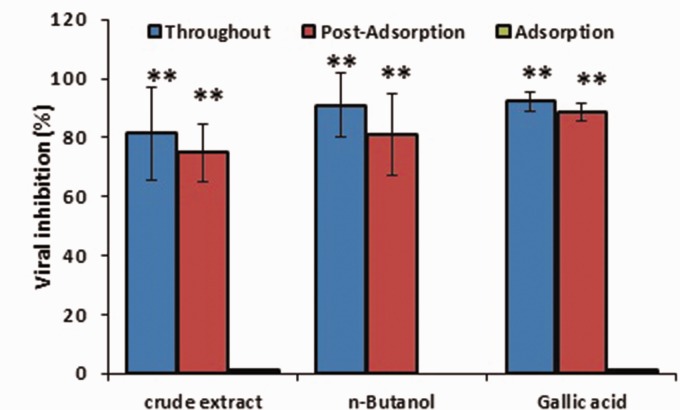
The effect of crude extract, *n*-butanol fraction, and gallic acid of pomegranate peel on the adsorption and post-adsorption of adenovirus. Data, expressed as mean ±standard error of measurement, show the percentage of virus inhibition compared with untreated control. One time 90% inhibitory concentration was used in this experiment. Statistical analysis was done by one-way analysis of variance followed by Tukey’s test. **p <* *0.001 for adsorption vs. post-adsorption and throughout.

Various experiments were carried out with one time IC_90_ of the plant materials with better anti-adenoviral effects to assess the mechanism of their antiviral action in the adsorption and post-adsorption phases of ADV replication (Figure 3). Our results showed that crude extract, *n*-butanol fraction and gallic acid inhibited ADV replication in the post-adsorption phase (*p *<* *0.01). There was no significant difference in the percentage of post-adsorption virus inhibition when these plant materials were present during all the experimental time (throughout phase; Figure 3).

## Discussion

In this study the inhibitory effect of crude extract, fractions, and main phenolic compounds of pomegranate peel on ADV in Hep-2 cell lines were investigated. Our results approved antiviral effect of pomegranate peel extract with IC_50_ of 5.77 µg/mL and SIs of 49.9. Among the fractions and compounds, the *n*-butanol fraction and gallic acid had the highest anti-adenoviral activity with an IC_50_ of 2.16 µg/mL and 4.67 µM and a SI of 122.5 and 10.5, respectively.

As the IC_50_ of the herbal extract against infectious diseases is less than 100 µg/mL,^16^ this extract and its *n*-butanol fraction should have strong activity against ADV.

Fifteen percent of pomegranate peel contains phenolic compounds including 8% ellagic acid, 3% punicalagin, and 0.8% gallic acid.^17^ According to previous studies, ellagic acid and gallic acid have anti-mutation, antiviral and antioxidant activities.^18^ In addition, punicalagin has been reported to exhibit a wide range of biological activities including antiviral effects on human immunodeficiency virus,^19^ influenza virus^20^ and herpes simplex virus.^21^

Because the anti-adenoviral effect of the pomegranate peel *n*-butanol fraction with a very high SI can be attributed to its main compounds including gallic acid, ellagic acid or punicalagin, the anti-adenoviral effects of these compounds were studied. The results of our study show that these compounds have a lower anti-adenoviral effect than the ethanolic fraction of pomegranate peel. Accordingly, it seems that the effect observed for the *n*-butanol fraction is related to the synergistic effects of the compounds present in this fraction, or other compounds. This argument needs to be further investigated.

Based on the results of this study, we suggest that in future studies, the amount of phenolic compounds in pomegranate peel fractions be evaluated using accurate methods such as high-performance liquid chromatography. Anti-adenoviral activity of punicalagin, combined with gallic acid, can also be evaluated to investigate its synergistic effect. We hope this study and supplemental research help to discover and produce effective compounds for the treatment of adenoviral diesis.

Various experiments were carried out with one time IC_90_ of the plant materials with better anti-adenoviral effects to assess the mechanism of their antiviral action in the adsorption and post-adsorption phases of ADV replication. Our results showed that crude extract, *n*-butanol fraction and gallic acid inhibited ADV replication in the post-adsorption phase. Based on our findings, the extract, *n*-butanol fraction, and gallic acid did not prevent the entry of ADV into Hep-2 cell but acted after penetration of the virus into the cell. This finding is in agreement with the results of other studies that have demonstrated medicinal plant extract reduces the viral titer when they are added after the adsorption phase.^15,22^

## Conclusion

Based on our results, pomegranate peel extract, especially its *n*-butanol fraction, could serve as a new promising anti-ADV agent due to its high inhibitory effect against ADV replication. The effect of the *n*-butanol fraction may be related to the synergistic effect or other compounds of this fraction. Further understanding of the action mechanism and the bioassay guided isolation of natural components of this fraction seems essential. More characterization of this extract probably leads to development of potential anti-adenoviral agents.
